# Cognitive Impairment in Adult Patients with 5q-Associated Spinal Muscular Atrophy

**DOI:** 10.3390/brainsci11091184

**Published:** 2021-09-09

**Authors:** Kathrin Kizina, Yakup Akkaya, Daniel Jokisch, Benjamin Stolte, Andreas Totzeck, Juan Munoz-Rosales, Andreas Thimm, Saskia Bolz, Svenja Brakemeier, Refik Pul, Derya Aslan, Jana Hackert, Christoph Kleinschnitz, Tim Hagenacker

**Affiliations:** Department of Neurology, University of Duisburg-Essen, 45147 Essen, Germany; yakup.akkaya@uk-essen.de (Y.A.); daniel.jokisch@uk-essen.de (D.J.); benjamin.stolte@uk-essen.de (B.S.); andreas.totzeck@uk-essen.de (A.T.); juan.munoz-rosales@uk-essen.de (J.M.-R.); andreas.thimm@uk-essen.de (A.T.); saskia.bolz@uk-essen.de (S.B.); svenja.brakemeier@uk-essen.de (S.B.); refik.pul@uk-essen.de (R.P.); derya.aslan@uk-essen.de (D.A.); jana.hackert@uk-essen.de (J.H.); christoph.kleinschnitz@uk-essen.de (C.K.); tim.hagenacker@uk-essen.de (T.H.)

**Keywords:** spinal muscular atrophy (SMA), motor neuron disease, cognition, intelligence quotient (IQ)

## Abstract

In previous studies, a below-average, average, or above-average intelligence quotient (IQ) in children with SMA was detected but, aside from a severe physical disability, the cognitive performance of adult SMA patients has not yet been evaluated. The intelligence test used in this study, the Wechsler Adult Intelligence Scale, fourth edition (WAIS-IV), was used to measure major intelligence components of adult SMA patients. The WAIS-IV determines four index scores representing verbal comprehension, perceptual reasoning, working memory, and processing speed. Due to time-dependent demands on motor function, the processing speed index score was excluded. IQ index scores of 33 adult SMA patients did not differ from IQ index scores of the normal population. In SMA type-3 patients, the index scores for verbal comprehension, perceptual reasoning, and working memory did not differ from the normal population but showed a trend of IQ scores towards lower points. Patients with SMA type 2 had lower IQ index scores for working memory (90.33 ± 12.95; *p* = 0.012) and perceptual reasoning (90.73 ± 12.58; *p* = 0.013) than the normal population. This study provided further evidence that SMA is a multi-systemic disease and may refute the widespread hypothesis that SMA patients might improve their cognitive skills to compensate for their physical impairment.

## 1. Introduction

Spinal muscular atrophy (SMA) is an autosomal-recessive disease that affects motor neurons in the anterior horn. It is a common genetic cause of early infant mortality with an incidence of 1:10,000, caused by homozygous disruption of the survival motor neuron 1 (SMN1) gene by mutation, deletion or conversion. This results in a defective and insufficient production of the SMN protein, which is an assembly protein for RNA-protein complexes that are critically involved in the survival and maintenance of motor neurons [1,2]. SMA leads to progressive muscle atrophy and weakness and can be distinguished in different types regarding the severity of symptoms, age of onset, and best achieved motor milestones. SMA type 0 is defined by a prenatal onset and characterized by restricted intrauterine movement [3]. Children with SMA type 1 (Werdnig–Hoffmann disease) depend on ventilatory support due to respiratory failure and never attain the ability to sit independently. Children with SMA type 2 learn to sit but never learn to walk unassisted. Patients with SMA type 3 (Kugelberg–Welander disease) learn to walk independently but may rely on a wheelchair during the course of their lives [4]. Irrespective of their SMA type, patients can also be classified as “non-sitters,” “sitters,” or “walkers,” which may vary over the course of life. This classification can be positively affected by successful treatment [5] or in a negative way by natural disease progression [1,6]. Tetraparesis can also be caused by other diseases. These include, for example, patients with a condition following an injury to the cervical spinal cord who have often also suffered a primary or secondary injury to the brain [7,8,9], and patients with amyotrophic lateral sclerosis (ALS), with manifestation in older patients [10,11].

Beside the progressive muscle weakness leading to challenges in daily life, SMA patients suffer also from fatigue and the feeling of depression [12,13]. Previous studies showed cardiac impairments, liver, pancreas, or intestinal dysfunctions, as well as metabolic deficiencies, especially in clinical severely affected children with SMA type 1 [14,15,16]. Hence, vulnerability to SMN deficiency may also not be confined to motor neurons due its ubiquitous expression. Anecdotally, it was often mentioned that children with SMA were smarter than healthy children of the same age. Clinicians have noted their keen interest in their surroundings, their mental acuity, and their observational abilities in relation to the very pronounced physical limitations [17,18]. It was speculated whether the development of knowledge and cognitive skills might be a creative method of compensating for their many restrictions [18,19]. Based on patient’s reports with SMA type 2 and 3, it is assumed that they might focus on different physical activities during their leisure time compared to patients without physical disability [17,18] for example reading books or listen to music instead of sports or physical activity. However, restricted sensorimotor interactions, as well as difficulties exploring objects and the environment, may cause cognitive impairment [20].

In previous studies of children and adolescents with SMA types 1–3, there was either a difference in the mean intelligence quotient (IQ) of the healthy population [21], intelligence was described as above [18] or below [20,22] average and in children with SMA type 0, severe brain involvement may likely be the full end manifestation of an already extreme phenotype [23]. However, there are currently very few systematic data on cognitive performance of adult patients with SMA.

Cognitive performance objectified as the intelligence quotient (IQ) can be measured in the fourth addition of the Wechsler Adult Intelligence Scale (WAIS-IV). David Wechsler published the original WAIS in February 1995. The latest fourth edition and most widely used IQ test for adults was released by Pearson in 2008 [24]. We chose the WAIS-IV because it is an internationally recognized and validated IQ test. In addition, most test subcategories do not include motor tests, which is particularly relevant to our patient cohort. For cognitive tests in children, previous studies used the adapted version of the Wechsler Test, the Wechsler Intelligence Scale for Children (WISC; German version: HAWIK-R) [18].

In this study, we compared IQ index scores of adult patients with SMA with normal population and between SMA groups using the WAIS-IV intelligence test.

## 2. Materials and Methods

The intelligence quotient (IQ) of adult patients with SMA was measured with the Wechsler Adult Intelligence Scale (WAIS). WAIS is currently in its fourth edition (WAIS IV) and the most widely used IQ test in adults between 16 and 89 years [25]. The IQ is a standardized score with a mean of 100 and standard deviation of 15 with respect to the normal distribution [26,27]. In this study, the German version of the WAIS-IV (released in 2012 by Petermann) was used, which was standardized in a sample of 1800 people [25]. The resulting full-scale IQ (FSIQ) consists of a verbal IQ (VIQ), a performance IQ (PIQ), and 10 core subtests. The VIQ was further subdivided into the Verbal Comprehension Index (VCI) with core subtests Similarities, Vocabulary and Information, and Working Memory Index (WMI) with core subtests Digit Span and Arithmetic. The PIQ was subdivided into the Perceptual Reasoning Index (PRI) with core subtests Block Design, Matrix Reasoning and Visual Puzzles, and Processing Speed Index (PSI) with core subtests Symbol Search and Coding. In addition to their core subtests, the four index scales include supplemental subtests that can be alternatively used [25]. The PSI was excluded due to time-dependent demands on the motor system. Because of different degrees of motor impairments, these tasks cannot provide valid measurements of the cognitive processing speed in these patients. Hence, an FSIQ consisting of all four components (VCI, WMI, PRI, and PSI) could not be measured in this study and we only focused on the VCI, WMI, and PRI. For the VCI and WMI, all of the core subtests were used. For the PRI, the core subtest Block Design was skipped due to demands on motor skills and replaced by the supplemental subtest Picture Completion. Regarding the test performance, every patient was instructed by the same investigator in a cross-sectional study from November 2018 to March 2019. All 33 patients received therapy with the antisense oligonucleotide (ASO) nusinersen. This study’s exclusion criteria were traumatic brain injuries, inflammatory brain diseases, mood disorder, psychiatric diseases, active or in the past, CNS affecting medication other than nusinersen, and other confounding factors that were possibly related to mental impairment. The influence of education level has only been descriptively analyzed, as the WAIS IV norm sample was also not subdivided according to educational level. In total, 34 patients were screened. One patient was screened and excluded before the beginning of the study because a craniocerebral trauma was reported several years ago.

In the classification and division of intelligence, 68% of people in a norm group had an IQ between 85 and 115. Statistical analyses and graph generation were conducted using SPSS version 22 (2013) and GraphPad Prism 9. The test scores of the SMA patients were compared with the normative dataset (mean ± standard deviation [SD]), normally distributed (100 ± 15), using one-sample *t*-test. Normality tests were performed before each *t*-test and *p* < 0.05 was considered statistically significant. A group comparison (SMA type 2 vs. SMA type 3) of the VCI, PRI, and WMI was performed by a *t*-test for independent measures.

## 3. Results

The 33 adult patients, 12 women and 21 men, included in this study had a mean age of 35 years (range 18–58 years). One patient was classified as SMA type 1 and was considered descriptively and only included in calculations of the total cohort. In total, 15 patients were classified as SMA type 2 and 17 patients as SMA type 3 (Table 1).

Of these 32 patients with SMA types 2 and 3, 21 (69%) graduated from high school and attained a university entrance qualification (SMA type 2, *n* = 11; and SMA type 3, *n* = 10). Of these 21 patients, 17 patients (53% of all 32 patients) currently study or studied at a university in Germany (SMA type 2, *n* = 10; and SMA type 3, *n* = 7). By comparison, in 2019, 34.6% of all school leavers in Germany attained a university entrance qualification [28].

**Figure 1:**
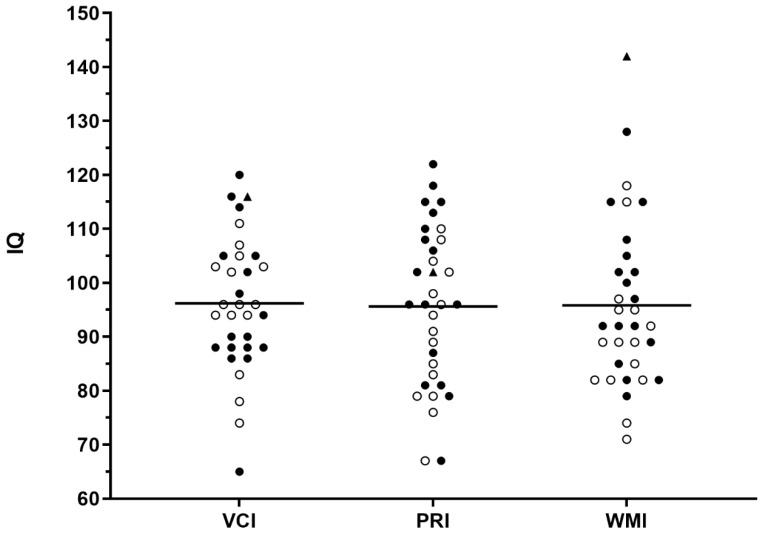
Intelligence quotient (IQ) for all patients. The IQ of the Verbal Comprehensive Index (VCI), Perceptual Reasoning Index (PRI), and Working Memory Index (WMI) subtests for each patient (*n* = 33) is shown. The mean of the normal population is 100 (SMA types 1: triangle, 2: unfilled circles and 3: filled circles).

Individual IQ scores of the intelligence domains VCI, PRI, and WMI were determined in the WAIS-IV. The cohort of 33 adult SMA patients had a mean IQ in the VCI of 96.21 ± 12.43, a median of 96 and with a difference in the means of −3.79 (95% CI: −8.2 to 0.62; *p* = 0.09, mean IQ in the PRI of 95.61 ± 14.76, a median of 96 and with a difference in means of −4.39 (95% CI: −9.63 to 0.84; *p* = 0.097), and mean IQ in the WMI of 95.82 ± 15.72, a median of 92 and with a difference in means of −4.18 (95% CI: −9.75 to 1.39; *p* = 0.136). The mean IQ of the SMA patients’ three different intelligence domains was not different from normal population (Figure 1).

**Figure 2:**
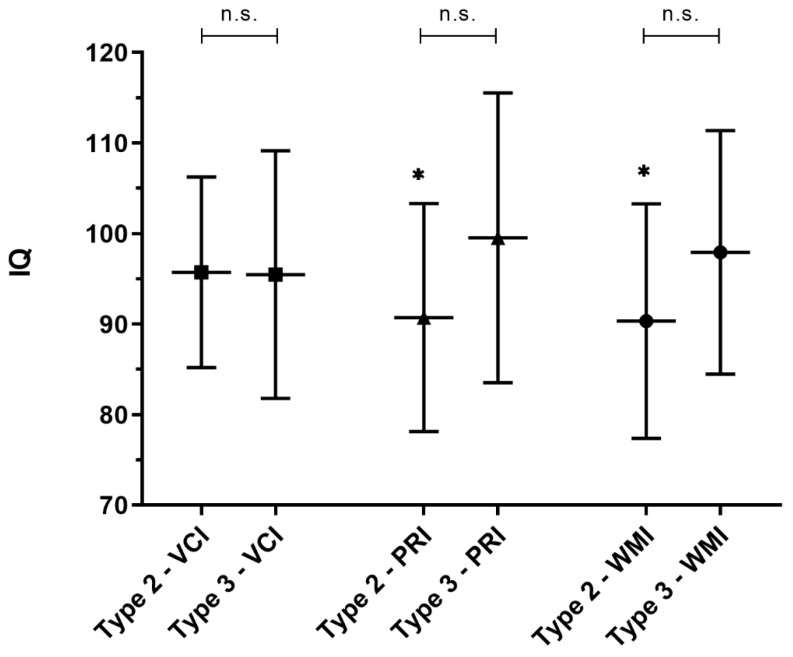
Intelligence quotient (IQ) of the patients with SMA types 2 and 3. The IQ of the VCI, PRI, and WMI subcategories of the patients with SMA type 2 (*n* = 15) and SMA type 3 (*n* = 17). The mean of normal population is 100. The IQ of the PMI and WMI of the patients with SMA type 2 are significantly reduced (*) compared to the normal population. The IQ of the patients with SMA type 2 compared to type 3 did not differ (n.s.) in the VCI (*p* = 0.95), PRI (*p* = 0.097), and WMI (*p* = 0.115).

The patients with SMA type 2 (*n* = 15) had a mean IQ in the VCI of 95.73 ± 10.52 (median: 96), in the PRI of 90.73 ± 12.58 (median: 91), and in the WMI of 90.33 ± 12.95 (median: 89). The mean IQ of the PRI and WMI was lower than the mean IQ (100) of normal population (PRI: difference in means of −9.27, 95% CI: −16.23 to −2.3; *p* = 0.013, effect size 0.62), (WMI: difference in means of −9.67, 95% CI: −16.84 to −2.5; *p* = 0.012, effect size 0.65). There was no difference in the IQ in the VCI compared to normal population (difference in means of −4.27, 95% CI: −10.09 to 1.56; *p* = 0.138) (Figure 2).

The IQ of the patients with SMA type 3 (*n* = 17) did not differ from the IQ of the normal population with a mean IQ in the VCI of 95.47 ± 13.67, a median of 90 and with a difference in means of −4.53 (95% CI: −11.56 to 2.5; *p* = 0.191), a mean IQ in the PRI of 99.53 ± 16.00, a median of 102 and with a difference in means of −0.47 (95% CI: −8.7 to 7.76; *p* = 0.905), and a mean IQ in the WMI of 97.94 ± 13.45, a median of 97 and with a difference in means of -2.06 (95% CI: −8.97 to 4.86; *p* = 0.537) (Figure 2). Nevertheless, IQ-scores in patients with SMA type 3 were lower compared to normal population data, but those differences were not statistically significant.

The IQ in the VCI, PRI, and WMI of the patients with SMA type 2 did not differ from the IQ in the three categories compared to the patients with SMA type 3 (VCI, *p* = 0.95; PRI, *p* = 0.097; and WMI, *p* = 0.115) (Figure 2).

**Figure 3:**
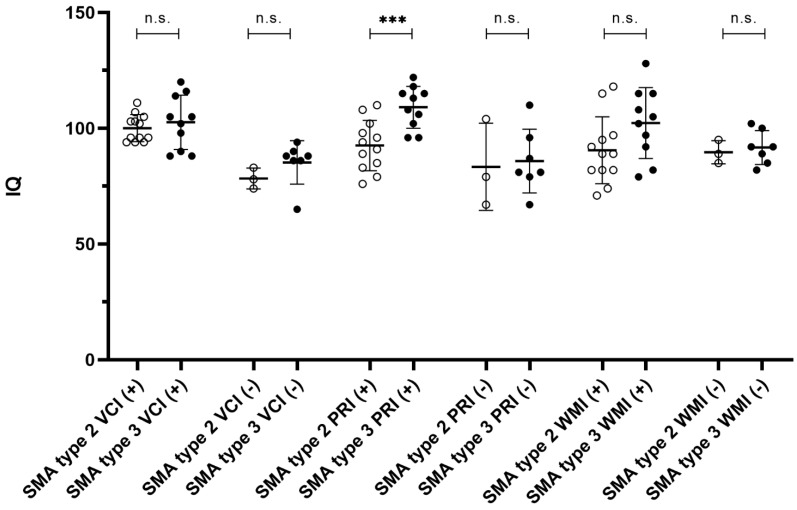
Intelligence quotient (IQ) and educational level. Intelligence quotient of the IQ subgroups (VCI, PRI, WMI) of patients with SMA type 2 (unfilled circles) and 3 (filled circles) subdivided into achieved university entrance qualification (+) and unachieved university entrance qualification (−). The IQ of the PMI in SMA type 2 patients with university entrance qualification is significantly reduced (***) compared to SMAP type 3 patients with university entrance qualification. The IQ of the other comparison groups did not differ (n.s.).

With regard to a correlation between university entrance qualification and IQ, we remain descriptive (Figure 3).

The patients with SMA type 2 and a university entrance qualification (*n* = 12) had a mean IQ in the VCI of 100.08 ± 5.82 (median: 99), in the PRI of 92.58 ± 10.86 (median: 93) and in the WMI of 90.50 ± 14.44 (median 89). The patients with SMA type 2 and no university entrance qualification (*n* = 3) had a mean IQ in the VCI of 78.33 ± 4.51 (median: 87), in the PRI of 83.33 ± 18.88 (median: 79) and in the WMI of 89.67 ± 5.03 (median: 89). The patients with SMA type 3 and university entrance qualification (*n* = 10) had a mean IQ in the VCI of 102.6 ± 11.69 (median: 104), in the PRI of 109.1 ± 9.04 (median: 111) and in the WMI of 102.3 ± 15.32 (median: 104). The patients with SMA type 3 and no university entrance qualification (*n* = 7) had a mean IQ in the VCI of 85.29 ± 9.36 (median: 88), in the PRI of 85.86 ± 13.77 (median: 81) and in the WMI of 91.71 ± 7.32 (median: 92).

Only the mean IQ of the PRI of patients with SMA type 2 and 3 with university entrance qualification differ from each other. It was lower in patients with SMA type 2 (difference in means of 16.52, 95% CI: 7.67 to 25.36, *p* = 0.001, effect size 1.64). The IQ of PRI of patients with SMA type 2 and 3 without a university entrance qualification did not differ from each other (difference in means of 2.52, 95% CI -36.17 to 41.22; *p* = 0.85). The mean IQ of the VCI of patients with SMA type 2 and 3 with and without university entrance qualification did not differ from each other (difference in means of 2.52, 95% CI −6.28 to 11.32; *p* = 0.55 and 6.95, 95% CI −3.27 to 17.18; *p* = 0.15). In addition, the mean IQ of the WMI of patients with SMA type 2 and 3 with and without university entrance qualification did not differ from each other (difference in means of 11.80, 95% CI −1.59 to 25.19; *p* = 0.08 and 2.05, 95% CI −7.89 to 11.99; *p* = 0.63).

In one patient with an SMA type 1 phenotype 6 SMN2 copies were detected. Regarding his cognitive performance, he had an extraordinary above-average IQ in the WMI of 142 and graduated from university. Because of these aspects, we considered him as outlier and did not include him in the statistical analysis.

## 4. Discussion

In this cohort of adult patients with SMA types 2 and 3, the IQ of the WMI and PRI subtests was lower in the patients with SMA type 2 than normal population. The IQ of the patients with SMA type 3 did not differ from normal population, nor did the IQ of the two SMA types 2 and 3 differ from each other. Because of severe motor disability and limitations in daily life caused by neuromuscular disorders, research so far have focused on pathophysiologic mechanisms of motor dysfunction, while cognitive performance has not paid much attention [29]. In dystrophinopathies the lack of different dystrophin isoforms also impairs CNS function [30]. In ALS frontal lobe dysfunction is a common manifestation beside motor neuronal degeneration [31].

Most studies so far on cognitive performance and impairment in SMA include only children or adolescents with SMA, although the progression of disease continues even into adulthood. One study also included adults but focused on SMA types rather than age [22]. Another recently published study focuses on the cognitive performance of adult SMA patients compared to patients suffering from ALS [32]. In previous studies, cognitive performance of children with SMA varies from average [21,33], above-average [18], and below-average [19,20,22]. In a study by Rivière et al. spatial cognition was in the normal range in patients with SMA type 2 compared to healthy children [33]. Above-normal cognitive performance in adolescents with SMA type 1-3 was determined in a study by von Gontard et al. [18]. In this study, some SMA type 1 patients between 6 and 18 years of age were still alive because their initial symptoms appeared only after 6 months. This made it possible to test cognition in older children and adolescents with SMA type 1, allowing a wider range of patients to be tested. Adolescents with SMA had a higher IQ than children with SMA, but there was no difference in IQ between the SMA types. Our cohort of adult SMA patients includes only one patient with SMA type 1 because the majority of patients with SMA type 1 do not reach adulthood due to the severity of the disease and respiratory insufficiency. Therefore, the calculation of the IQ of adult patients with SMA type 1 is limited. In a study by Polido et al., children with SMA type 1 were tested compared to healthy children of the same age [20]. Cognitive performance in SMA children was below normal. In this study, as well as in our study, it was discussed whether cognitive impairment would be primary neurodegenerative or secondary developmental. It is not yet clear whether the SMN2 deficit in the central nervous system primarily causes a lower IQ, or whether patients are not able to explore their surroundings due to muscle weakness and, therefore, their IQ cannot develop fully. Chung et al. studied SMA patients from 1 to 40 years [22]. Cognition scores of SMA patients, using the WeeFIM, were below normal and scores of SMA type 1 patients were lower than scores of patients with types 2 and 3. In a study comparing the cognitive performance of SMA patients with patients suffering from ALS, the Edinburgh Cognitive and Behavioural ALS Screen (ECAS) was used. SMA patients achieved better results in terms of cognitive performance compared to the ALS patients [32]. ALS, similar to SMA, is a motor neuron disease, but with profound differences in pathophysiology. Cognitive deficits are part of the systemic neuronal degeneration and not a secondary aspect. In addition, the mean age of onset in ALS patients is around 60 years. Until then, these patients also usually go through a normal development, so that a comparison may distract here [10,11]. Overall, an ideal control group may be infeasible to recruit. In all of the aforementioned studies, different tests and patient cohorts were used, partially with or without control groups. In some cases, no genetic testing was conducted, so the diagnosis of SMA was not validated [22,33]. In our study, the IQ of the adult SMA patients was compared with the IQ of normal population. The VCI (as part of the VIQ) of the SMA type 2 patients did not differ from normal population, which may support the hypothesis that attempts could partly be made to compensate for the motor deficits caused by SMA. As previously discussed, SMA might be related to the theory that the lack of SMN protein not only affects spinal motor neurons but also other cellular compartments of the central nervous system [23]. This might lead to the hypothesis that patients with a lower SMN2 copy number and thus less SMN proteins are also clinically more severely affected and consequently have a lower IQ. In our cohort, most of the patients have 3 or 4 SMN2 copy numbers. Because of the small number of patients included in this study, we classified them according to SMA types and not according to SMN2 copy numbers, patients age, educational level and duration or onset of disease to prevent a further reduction in sample size. Due to the small sample size of SMA patients with and without university entrance qualification, only a descriptive analysis could be performed, which also minimizes selection bias. Therefore, a correlation between educational level and IQ subgroups of SMA types could not be investigated in detail. One may hypothesize that cognitive deficits could have developed due to a deficiency in the SMN1 protein in the CNS, and patients with SMA type 2 and a more severe disease course would have a lower probability of attaining a university entrance qualification. In addition, we did not specifically analyze the cohort gender, as the healthy control group in WAIS IV also did not determine IQ gender-specifically. In our cohort, no further test adjustments regarding age were necessary as it was in children in previous studies because of their different state of development. Therefore, all of the patients engaged in the same tasks and the comparison of the test results might be less prone to errors [25].

All of our patients were treated with nusinersen, but we do not expect a change in cognitive performance during therapy because, in animal experiments, an intrathecal injection led to a high enrichment and distribution of nusinersen in the spinal cerebrospinal fluid and thoracic and lumbar spinal cord. In brain cells (cerebellum, pons, and cortex), only very low concentrations were measured [34,35]. However, a nusinersen effect cannot be fully excluded. As most adult patients are now on treatment, untreated cohorts might be hard to recruit.

Due to the impaired motor skills, the WAIS-IV was chosen for this study because of the few motor tests and the focus on the IQ. FSIQ could not be calculated because the index containing processing speed (PSI) was skipped to create equal conditions for all of the patients. For the same reason, the Block Design subtest in the PRI category was skipped for all of the patients and, instead, the supplemental Picture Completion subtest was used. Therefore, only the IQ of the three index scales (VCI, PRI, and WMI) could be compared with the normal population. The concept of IQ is independent of age, gender or schooling, or the standard values are scaled accordingly. Therefore, a further division of patients into groups regarding to age, sex or years of education was not necessary to perform the WAIS-IV [25]. For longitudinal trajectories especially under disease-modifying treatment of early-onset SMA, repeated IQ testing will be of interest. To prevent from the influence of training effects, the WAIS-IV offers different versions. Therefore, simplified cognitive performance tests, such as MOCA or MMSE, are not feasible, especially since these tests are also validated for use in dementia or other neurodegenerative diseases and are mainly used as screening tools [36,37].

## 5. Conclusions

In this cohort of adult SMA types 2 and 3 patients, the IQ index scores for Working Memory and Perceptual Reasoning were lower in the patients with SMA type 2 than the normal population. The IQ index scores of SMA type 3 patients were not different from the normal population, but there was a trend towards lower cognitive performance. This study provided further evidence that SMA is a multi-systemic disease and may refute the widespread hypothesis that SMA patients might improve their cognitive skills to compensate for their physical impairment.

## Figures and Tables

**Table 1:** Patients.

Characteristics	Total	SMA Type 1	SMA Type 2	SMA Type 3
	(*n* = 33)	(*n* = 1)	(*n* = 15)	(*n* = 17)
	No. (~%)	No. (~%)	No. (~%)	No. (~%)
Female	12 (36)	0	9 (60)	3 (18)
Male	21 (64)	1 (100)	6 (40)	14 (82)
Age (years, mean ± SD (range))	35 ± 11 (18–58)	29	31 ± 10 (18–48)	38 ± 12 (19–58)
SMN2 copy number				
3	19 (58)	-	14 (93)	5 (29)
4	11 (33)	-	1 (7)	10 (59)
5	1 (3)	-	-	1 (6)
6	1 (3)	1 (100)	-	-
7	1 (3)	-	-	1 (6)
Ambulatory	8 (24)	-	-	8 (47)
Non-ambulatory	25 (76)	1 (100)	15 (100)	9 (53)
University entrance qualification	22 (67)	1 (100)	11 (73)	10 (59)
Enrolled at/graduated from university	18 (55)	1 (100)	10 (67)	7 (41)

Note. The patient with SMA type 1 was mentioned in this table but not included in the statistical analysis.

## Data Availability

The data presented in this study are available on request from the corresponding author. The data are not publicly available due to privacy reasons.
